# Tunable Blood Shunt for Neonates With Complex Congenital Heart Defects

**DOI:** 10.3389/fbioe.2021.734310

**Published:** 2022-01-13

**Authors:** Ellen Garven, Christopher B. Rodell, Kristen Shema, Krianthan Govender, Samantha E. Cassel, Bryan Ferrick, Gabriella Kupsho, Ethan Kung, Kara L. Spiller, Randy Stevens, Amy L. Throckmorton

**Affiliations:** ^1^ BioCirc Research Laboratory, School of Biomedical Engineering, Science, and Health Systems, Drexel University, Philadelphia, PA, United States; ^2^ Tissue Instructive Materials Laboratory, School of Biomedical Engineering, Science, and Health Systems, Drexel University, Philadelphia, PA, United States; ^3^ Biomaterials and Regenerative Medicine Laboratory, School of Biomedical Engineering, Science, and Health Systems, Drexel University, Philadelphia, PA, United States; ^4^ Department of Mechanical Engineering and Bioengineering, Clemson University, Clemson, SC, United States; ^5^ Pediatrics, College of Medicine, Drexel University, Philadelphia, PA, United States; ^6^ Heart Center for Children, St. Christopher’s Hospital for Children, Philadelphia, PA, United States

**Keywords:** blood flow, computational fluid dynamics, biomaterials, hydrogels, pediatrics, single ventricle physiology, shunt, congenital heart defects

## Abstract

Despite advancements in procedures and patient care, mortality rates for neonatal recipients of the Norwood procedure, a palliation for single ventricle congenital malformations, remain high due to the use of a fixed-diameter blood shunt. In this study, a new geometrically tunable blood shunt was investigated to address limitations of the current treatment paradigm (e.g., Modified Blalock-Taussig Shunt) by allowing for controlled modulation of blood flow through the shunt to accommodate physiological changes due to the patient’s growth. First, mathematical and computational cardiovascular models were established to investigate the hemodynamic requirements of growing neonatal patients with shunts and to inform design criteria for shunt diameter changes. Then, two stages of prototyping were performed to design, build and test responsive hydrogel systems that facilitate tuning of the shunt diameter by adjusting the hydrogel’s degree of crosslinking. We examined two mechanisms to drive crosslinking: infusion of chemical crosslinking agents and near-UV photoinitiation. The growth model showed that 15–18% increases in shunt diameter were required to accommodate growing patients’ increasing blood flow; similarly, the computational models demonstrated that blood flow magnitudes were in agreement with previous reports. These target levels of diameter increases were achieved experimentally with model hydrogel systems. We also verified that the photocrosslinkable hydrogel, composed of methacrylated dextran, was contact-nonhemolytic. These results demonstrate proof-of-concept feasibility and reflect the first steps in the development of this novel blood shunt. A tunable shunt design offers a new methodology to rebalance blood flow in this vulnerable patient population during growth and development.

## Introduction

Each year, approximately 4 million babies are born in the United States, 1% of which have a congenital heart defect requiring intervention (Oster et al., 2014; Mozaffarian et al., 2015). A subset of these infants contend with substantial structural anomalies characterized as single ventricle (SV) physiology, such as tricuspid atresia or hypoplastic left heart syndrome (HLHS). SV defects occur in approximately 1,000–2,000 live births annually (Reller et al., 2008). In contrast to normal cardiovascular physiology with two pumping chambers or ventricles, an SV physiology consists of only one functional ventricle to drive and draw blood through both the systemic and pulmonary circulations. This anatomy results in limited perfusion, continual mixing of oxygenated and deoxygenated blood within the SV, and 100% mortality without surgical intervention.

**FIGURE 1:**
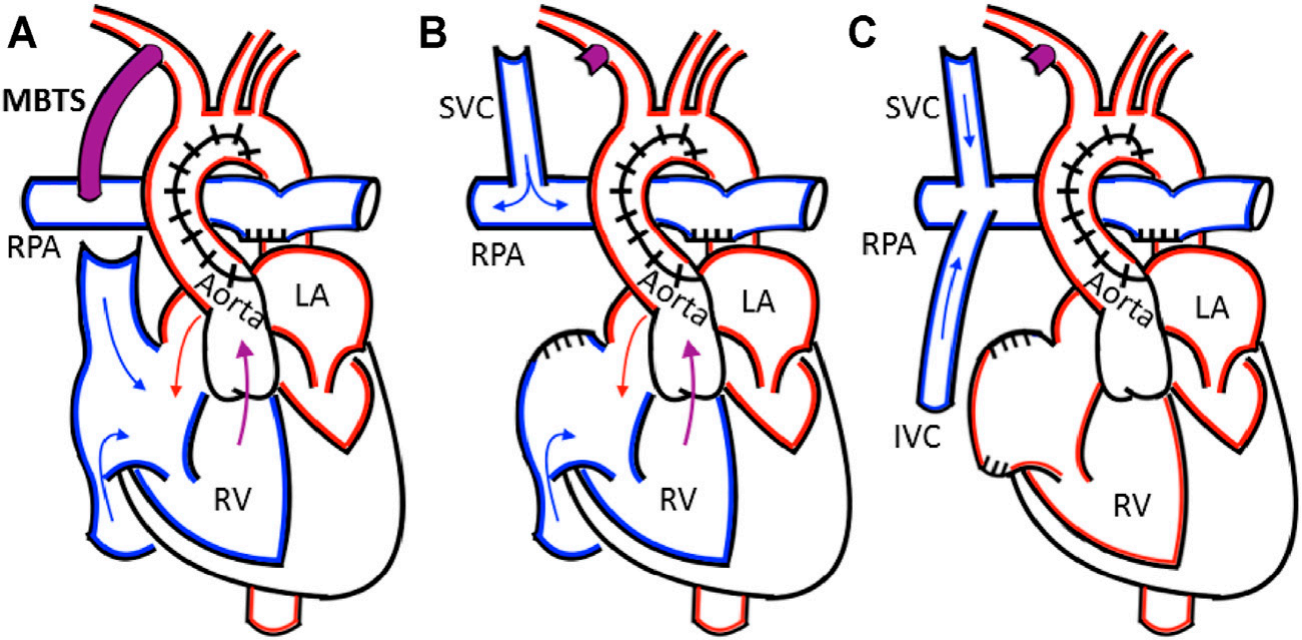
**(A)** The Norwood procedure with a Modified Blalock-Taussig shunt (MBTS) from the innominate into the right pulmonary artery **(B)** In the bidirectional Glenn, the shunt is ligated and the superior vena cava is attached to the right pulmonary artery **(C)** The Fontan procedure attaches the inferior vena cava to the pulmonary artery, creating the total cavopulmonary connection. The relevant anatomy includes the right pulmonary artery (RPA), right ventricle (RV), left ventricle (LV), left atrium (LA), superior vena cava (SVC), and inferior vena cava (IVC).

**FIGURE 2:**
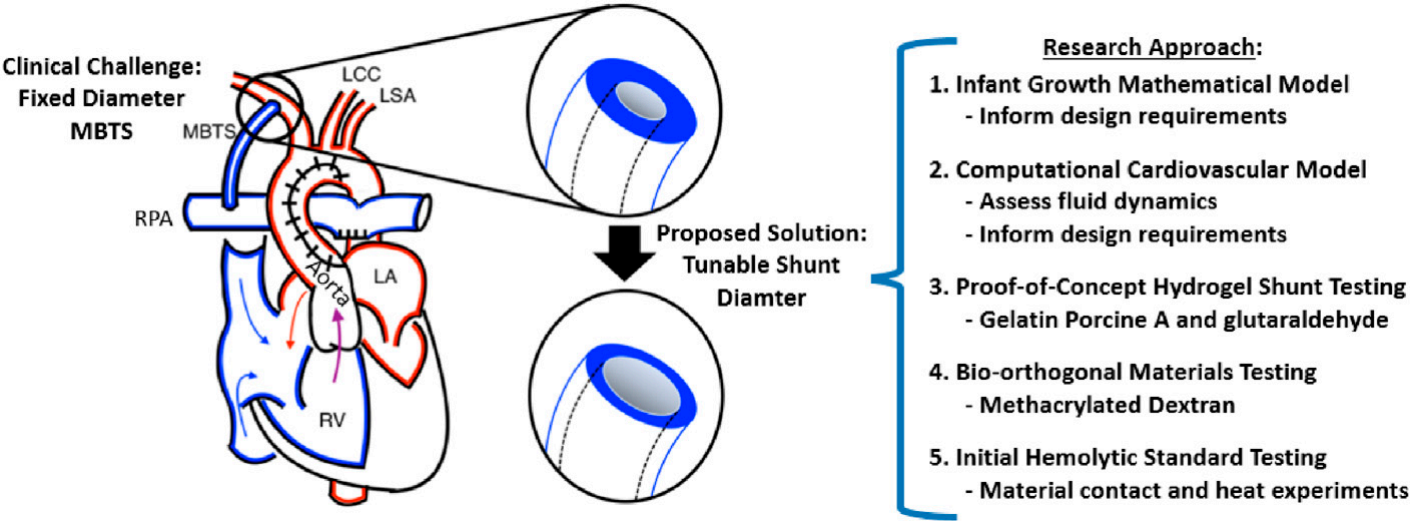
Overview of the Study. The Modified Blalock-Taussig shunt (MBTS) implanted in a single ventricle heart connects the systemic and pulmonary circulations, with the relevant anatomy including the right pulmonary artery (RPA), right ventricle (RV), left ventricle (LV), left atrium (LA), left common carotid (LCC), and left subclavian (LSA). We developed an infant growth mathematical model and computational cardiovascular model, which both informed the design requirements for the proof-of-concept hydrogel prototype testing and then bio-orthogonal prototype testing with initial hemolytic standard experiments.

The current treatment (Figure 1) is a palliative, three-staged, open-heart surgical sequence (Reller et al., 2008; Ohye et al., 2010). In the first stage, the Norwood procedure, the single functional ventricle is connected to both the systemic and pulmonary circulations via the implantation of a blood shunt, which directs blood flow into the pulmonary arteries from the systemic circulation. The shunt remains in place for 3–6 months until the second stage procedure (Ohye et al., 2010). There are several shunt configurations; conventional procedures most commonly use the Modified Blalock-Taussig shunt (MBTS, Figure 2) configuration, where the GORE^®^ PROPATEN^®^ Vascular Graft configured for pediatrics is the preferred shunt. It is comprised of an expanded polytetrafluoroethylene (ePTFE) tube that is surface-modified by heparin to minimize thrombogenicity.

Norwood patients require a delicate balance of blood flow to each of the parallel systemic and pulmonary circulations. Complications frequently result from instabilities in the systemic-to-pulmonary blood flow, either hyperperfusion or hypoperfusion of the pulmonary arteries as delivered through the shunt. A blood shunt diameter that is too narrow has an increased internal resistance, which may lead to pulmonary hypoperfusion, higher shear stresses, and irregular blood flow patterns that boost the risk of hemolysis and thrombosis. On the contrary, a shunt that is too large draws more blood volume to the lungs and away from the systemic circulation, adversely impacting the oxygenation of systemic end organs. Thus, it is critically important to select an appropriate shunt size to balance flow distribution.

While the shunt diameter is carefully chosen, many other surgical and non-surgical factors can impact the balance of blood flow between the lungs and body. Shunt length and orientation, vessel size, cardiac output, and pulmonary vascular resistance can all alter the systemic-to-pulmonary blood flow as the patient develops. Clinicians manage the systemic-to-pulmonary blood flow balance using pharmacological therapies, percutaneous interventions, or surgical correction. While pharmacological management is routine, physical intervention (including dilation, stent placement, or surgical correction) has been reported in as many as 47.9% of MBTS cases between the Norwood and Stage 2 surgergy (Ohye et al., 2010). Mortality rates (7–19%) for the Norwood remain among the highest in cardiac surgery and have not improved in decades despite advancements in clinical procedures and patient care (Ohye et al., 2010; Petrucci et al., 2011; Ohye et al., 2012). The high risk of complications and the lack of sustained improvements in clinical outcomes highlight a fundamental flaw in using a fixed diameter blood shunt. A shunt geometry that is specifically designed to facilitate control over the balance of systemic-to-pulmonary blood flow during developmental growth would be a strong advance.

To address this unmet clinical need, we have developed a geometrically tunable, hydrogel-lined conduit for use as a blood shunt. This shunt was designed to have an expandable inner lumen diameter that can be controllably modified in proportion to the growth and development of the patient. Thus, this offers a new approach to intrinsically adjust the ratio of systemic to pulmonary blood flow. The device design includes a fixed-diameter outer sheath coated internally with a thick hydrogel layer. Hydrogels are water-swollen polymer networks, known for their biocompatibility and anti-fouling properties that make them ideal candidates for blood-contacting biomaterials, such as catheters and shunts. Their degree of crosslinking also directly controls hydrogel swelling, which means that increasing the degree of crosslinking decreases swelling and overall dimensions. Thus, we reasoned that a hydrogel lining of a blood shunt would shrink as crosslinking is increased, resulting in an overall wider inner lumen diameter if the other diameter is fixed, allowing the use of hydrogel crosslinking to control blood flow. This aspect of the hydrogel structure could be controlled by the infusion of crosslinking agents or by near-UV photoinitiation.

**FIGURE 3:**
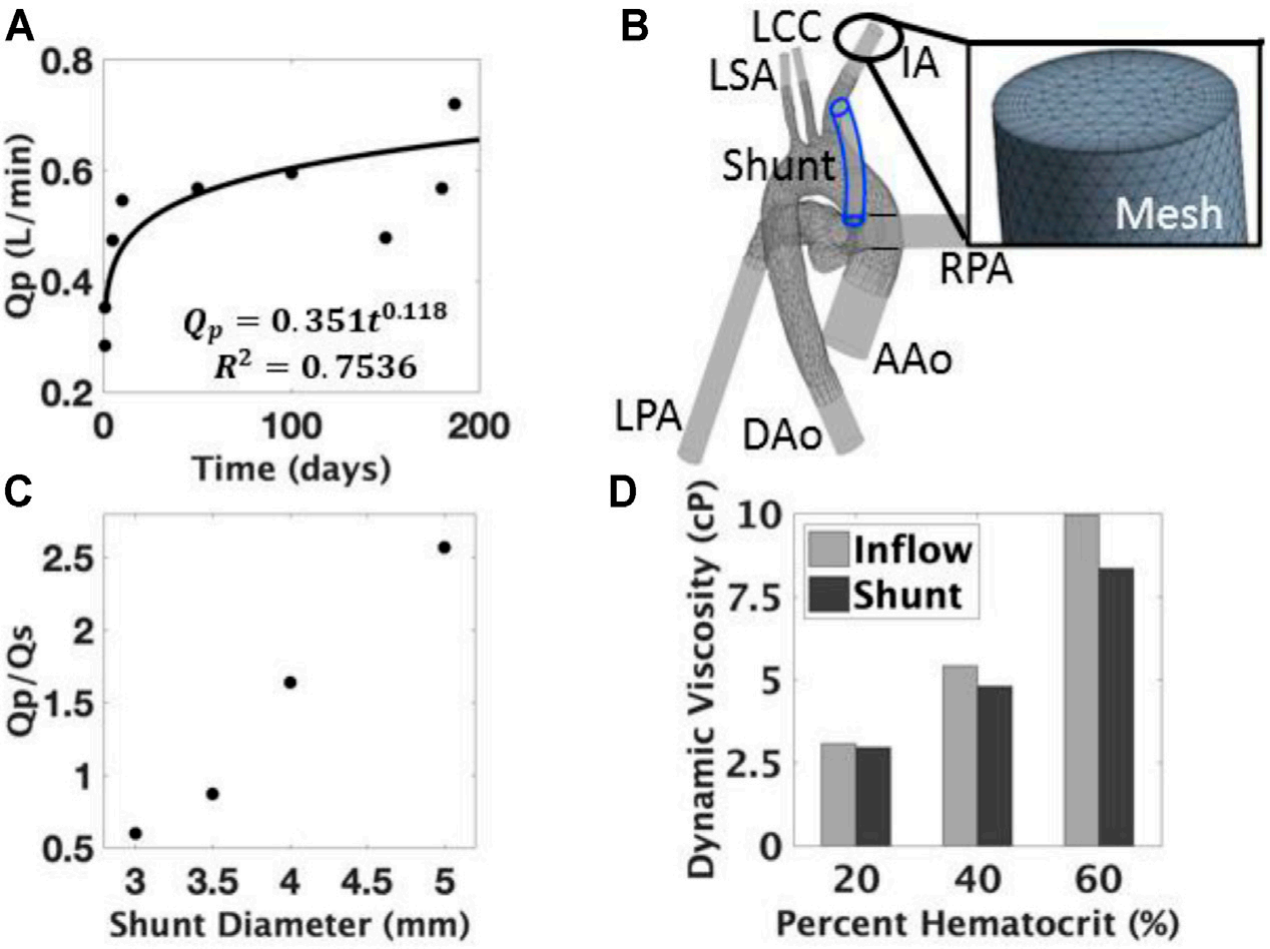
**(A)** Pulmonary blood flow (Qp) model during growth and development; **(B)** 3D anatomical computational model was developed to include the ascending aorta (AAo), descending aorta (DAo), innominate artery (IA), left common carotid (LCC), left subclavian (LSA), left pulmonary artery (LPA), and right pulmonary artery (RPA); **(C)** The effect of diameter on the Qp/Qs ratio showed an increased amount of pulmonary flow with larger diameters. **(D)** The effects of the viscoelastic model are measured through the changes in viscosity at varying locations, the difference of which widens with respect to percent hematocrit.

In this study (Figure 2), mathematical and computational fluid dynamic (CFD) cardiovascular models (Figure 3) were established to investigate the hemodynamic requirements of growing neonatal patients with shunts and to inform design criteria for shunt diameter changes. Then, two stages of prototyping were performed to design, build and test responsive hydrogel systems that allow the shunt diameter to be tuned according to the hydrogel’s degree of crosslinking. We first formed prototypes from a simple hydrogel system of gelatin further crosslinked by glutaraldehyde, and we sought to explore the feasibility of using hydrogel crosslinking to widen inner lumen diameter while the outer diameter remained fixed. In the second set of hydrogel experiments, we employed more biocompatible materials while also utilizing bio-orthoginal photocrosslinking chemistries to enable eventual use within the body. We fabricated these hydrogel prototypes using methacrylated dextran (DexMA) hydrogels having various degrees of methacrylate modification, photocrosslinked in the presence of a water-soluble and cytocompatible photoinitiator (lithium phenyl-2,4,6-trimethyl-benzoyl-phosphinate; LAP) that has broad absorbance including the near-UV range, which would allow initiation of crosslinking in a patient via fiberoptic illumination. All hydrogel prototypes were assessed for dimensional changes over time. Finally, we tested the contact hemolytic potential of methacrylated dextran-based hydrogel according to ASTM standard F756-17. Static contact experiments are critical first steps in proof-of-concept studies like these. These investigations demonstrate strong proof-of-concept and lay the groundwork for the translational development of this new tunable blood shunt.

## Materials and Methods

### Growth Mathematical Model

In order to estimate a target dimensional change for the inner lumen diameter of the tunable shunt, we had to establish an infant growth model. To our knowledge, no such growth model for Norwood recipients exists. We gathered available growth data (Emmanouilides et al., 1995; Throckmorton et al., 2005), especially measurement data of pulmonary blood flow (Qp). These data were fit with a power law regression model (Figure 3A), which allowed for the characterization of blood flow requirements for these patients over time (i.e. during growth). Using the Hagen-Poiseuille model (Eq. 1) for blood flow in a tube, we estimated the range of inner lumen diameter modulation that would be required to support infant growth and development.

$$Q=\frac{r^{4}\pi \left(\text{Δ}P\right)}{8\mu L}$$ (1)

where r signifies the radius, ΔP is the pressure drop across the shunt, μ refers to the dynamic blood viscosity, L is the shunt length, and Q is the blood flow through the shunt (i.e., pulmonary blood flow, Qp). The power-law regression model was then set equal to the Hagen-Poiseuille relationship (Eq. 1) so that we could determine a range of viable shunt diameters. This enabled us to set target requirements for the change in inner lumen diameter over time in the hydrogel experiments.

### Computational Cardiovascular Modeling

To evaluate the hemodynamic effect of changes in the shunt diameter, a model for CFD analysis was developed. Publicly available MRI imaging data of a healthy adult were utilized (Medical Connections Ltd., United Kingdom) and processed into a three-dimensional model using Mimics (Materialize, Leuven, Belgium). The anatomy of the aortic arch and pulmonary artery was isolated and imported into Solidworks (Dassault Systems, Waltham, Massachusetts, United States), where the model was scaled to a neonatal size (body surface area of 0.2 m^2^) based on typical dimensional data (Machii and Becker, 1997; Dodge-Khatami et al., 2005; Garcia-Canadilla et al., 2014; Park, 2014). An MBTS shunt was integrated between the innominate artery and right pulmonary artery, the placement and geometry of which was approved by an experienced pediatric cardiothoracic surgeon.

ANSYS 15.0 CFX (ANSYS Inc., Canonsburg, PA, United States) was employed to mesh the geometry (Figure 3B). A mesh size of approximately 6 million elements was developed after a grid independence study was conducted; a range of grid densities monitored at several locations were evaluated for <3% variation in velocities values and pressure differentials. The mesh was further refined to satisfy the dimensionless wall distance (y+ < 1) requirement for the κ-ω turbulence model, and to confirm mesh quality metrics including the skewness (max <0.95, average <0.33) and aspect ratio (max <200, average <50). A parabolic velocity profile using the standard one-seventh power law was implemented as the inflow boundary condition at the ascending aorta. The inlet profile was set to an average flow rate of 0.9 L/min, which reflects the average neonatal ventricular output before ductus closure (Pekkan et al., 2008). Preliminary simulations were conducted to determine effective pressures at the outlets representative of the downstream resistances in steady state. These outflow boundary conditions were set to obtain a physiological distribution of flow to each vessel, by matching to reported average flow splits (Lagana et al., 2005). All simulations were conducted under steady-state conditions, with a no-slip condition on all surfaces and a high-resolution advection scheme. Convergence criteria were satisfied when the maximum residual error reached less than 10^–3^.

Four shunt diameters were computationally evaluated (3.0, 3.5, 4.0, and 5.0 mm), corresponding to the range of available clinical options per our surgical collaborator. For these studies, blood was assumed to be Newtonian with a dynamic viscosity of 3.5 cP and density of 1,050 kg/m^3^. Although blood is known to be non-Newtonian, it is often modeled to have Newtonian behavior for shear rates greater than 100 s^−1^ (Al-Azawy et al., 2017).

To simulate the potential impact of “overgrowing” the shunt, we created another geometry based on the inner lumen diameter target. The identified target was implemented in a geometry based on a percent increase from an initial diameter of 3.5 mm. This geometry was simulated with a cardiac output proportional to the flow growth model (Figure 3A) and effective pressures that preserved the targeted flow distributions. These boundary conditions were then applied to the 3.5 mm model to examine the hemodynamics of a shunt left in place during physiological changes due to growth.

In another diameter comparison, a multiscale model was established and implemented, in which the boundaries of the CFD model were coupled to a lumped parameter network (LPN) of the Norwood physiology. The LPN uses an electrical analogy to represent the fluidic system in a set of ordinary differential equations, the solution of which is approximated with a 4th order Runge-Kutta method. The LPN and CFD models are simulated in parallel; at every timestep, each model solves for the pressures and flows needed as boundary conditions in the other model. The LPN was adapted from the work of Kung et al., and has a rich history of publication and validation (Kung et al., 2013; Kung et al., 2014; Schmidt et al., 2017). This model was used to recreate two diameters in the previous study, 3.0 and 3.5 mm, the data of which will be compared to the CFD-only results for an evaluation of the methodology.

We also evaluated the effect of a viscoelastic blood model, and we assessed which regions were associated with low shear rates and determined the suitability of the Newtonian assumption. A viscoelastic blood model was implemented based on the work of Good et al. (2016), with three hematocrits (20, 40, and 60%) covering the full range expected clinically.

Simulation results were assessed qualitatively and quantitively. Streamlines were inspected for vortices and eddies. The ratio of pulmonary to systemic flow (Qp/Qs) was calculated as a measure of clinical success in balancing blood flow. The average and maximum velocities, shear rates, and shear stresses were gathered for comparison. The diameter simulations were compared against previous literature reports.

### Initial Gelatin Prototype

**FIGURE 4:**
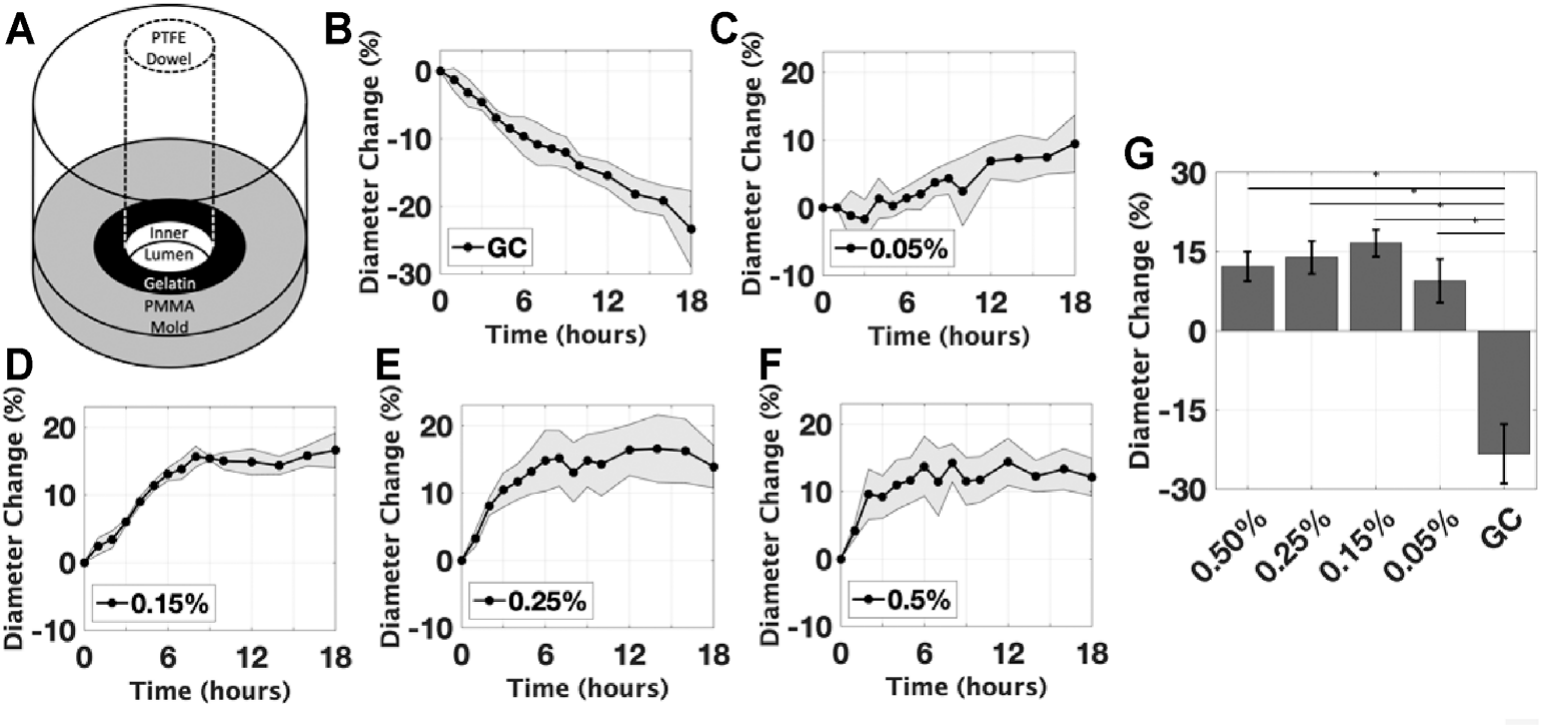
Initial Gelatin Experiments. **(A)** A schematic of the mold with dowel used to gelate the prototype; The effect of glutaraldehyde concentration on the mean inner diameter expansion, for concentrations of **(B)** control group without glutaraldehyde, **(C)** 0.05%, **(D)** 0.15%, **(E)** 0.25%, **(F)** 0.5%. **(G)** The diameter change measured at the final timepoint of each group is compared, all groups were found to be significantly different than GC (*p < 0.05). Data are reported as mean ± s.d.

To construct the first hydrogel prototype, a circular annular (ring) mold was created using poly(methyl methacrylate) (PMMA) with an inner polytetrafluoroethylene (PTFE) dowel that was placed in the center and formed the inner lumen of the casing. The PMMA surface was chemically modified by aminolysis with 1 M ethylenediamine-DMSO solution (Brown et al., 2006). This added amine functional groups, to ensure that the outer diameter of the hydrogel layer remained fixed to the PMMA ring following crosslinking. Gelatin (porcine skin, type A) solutions were prepared between 4–12% weight/volume (w/v), poured into the mold, and allowed to crosslink during cooling. Then, the interior dowel was removed, resulting in a hydrogel annulus (Figure 4A). We concentrated on the inner diameter changes of the hydrogel prototypes for this initial study; the axial length changes were not focused on during this study. The ring thickness or axial depth averaged approximately 1–2 cm.

### Effects of Crosslinking Density

**FIGURE 5:**
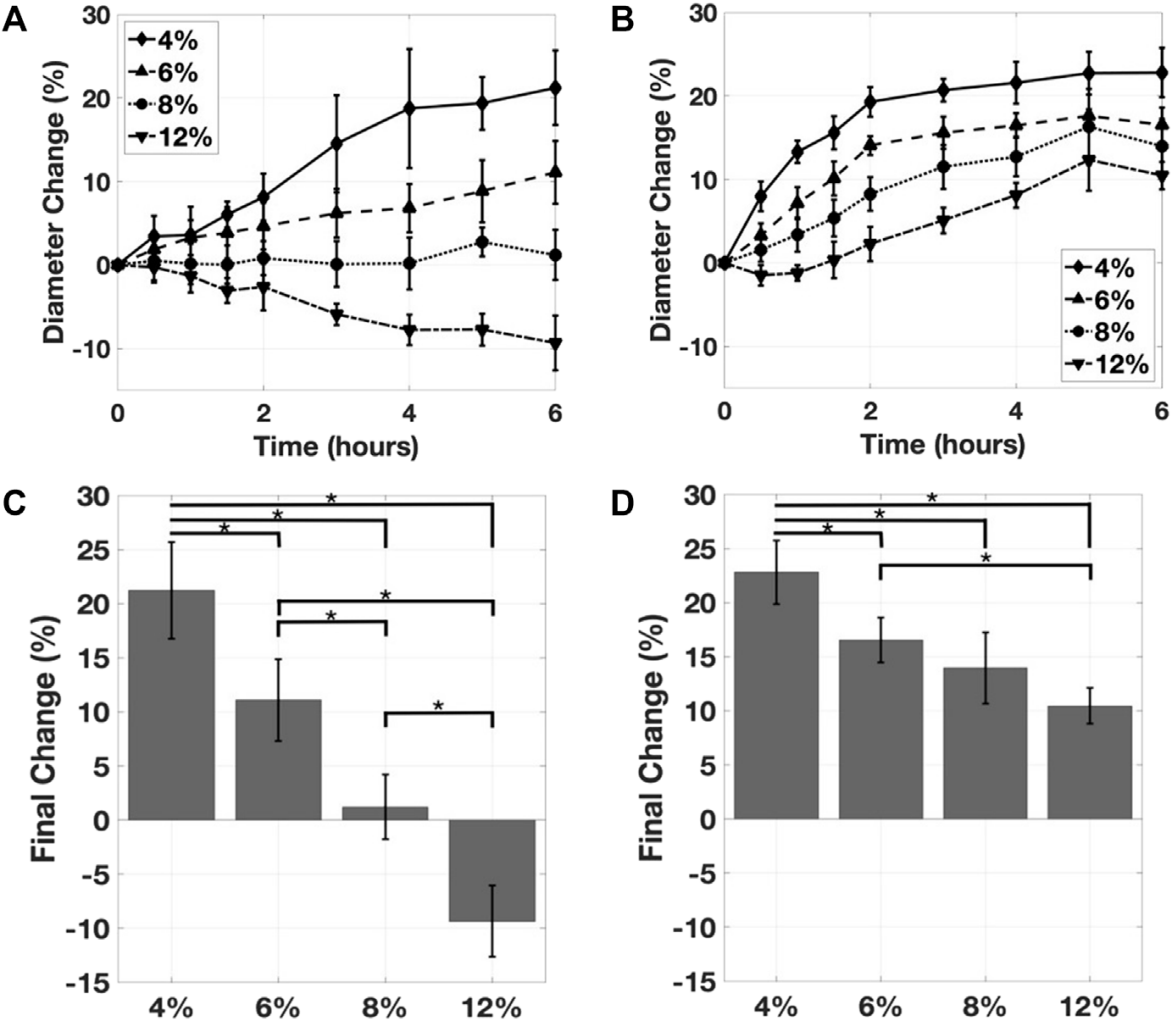
**(A)** The effect of gelatin concentration on the mean inner diameter expansion, with 0.05% glutaraldehyde. **(B)** The effect of gelatin concentration on the mean inner diameter expansion, with 0.50% glutaraldehyde. The effect of gelatin concentration on the inner diameter expansion as measured at the timepoint, for **(C)** the 0.05% glutaraldehyde and **(D)** the 0.50% glutaraldehyde samples. Mean ± s.d.; *p < 0.05.

Initially, the effect of crosslinker concentration on the inner diameter of the prototype was evaluated (Figure 5). For proof-of-concept, we chose gelatin-based hydrogels and the crosslinking agent glutaraldehyde because these are readily available materials with well-characterized interactions. The hydrogel was prepared using a 10% w/v gelatin solution in phosphate-buffered saline (PBS) and was left to reach equilibrium for 48 h. Then, to achieve increasing degrees of crosslinking, the hydrogels were immersed in a bath of PBS and a targeted concentration of glutaraldehyde, a known crosslinker of gelatin at room temperature (Chatterji, 1989). The hydrogel prototypes were treated by glutaraldehyde at varying concentrations (0, 0.05, 0.15, 0.25, and 0.50%, *n* = 6 per group) for 18 h. The effect of the gelatin concentration was likewise evaluated. Hydrogels were prepared using 4, 6, 8, and 12% w/v gelatin solutions and submerged in glutaraldehyde solutions (0.05 and 0.50%, *n* = 6 per group). For all conditions, samples were photographed at regular time intervals to capture the diameter changes.

### Methacrylation of Dextran

**FIGURE 6:**
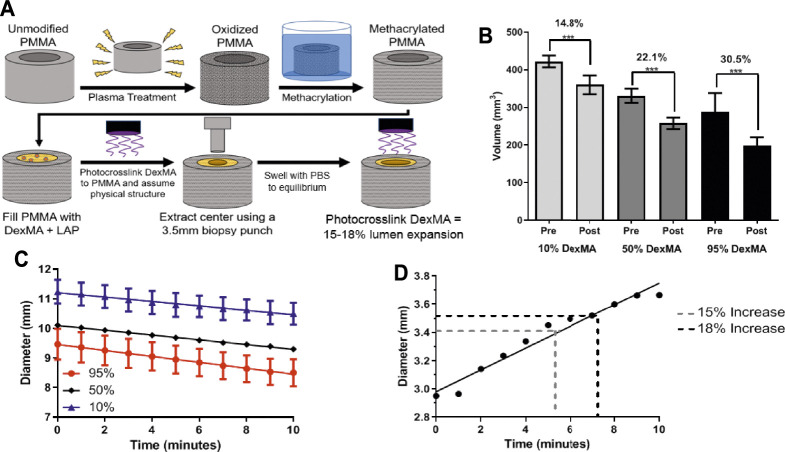
**(A)** A schematic of the PMMA tubing functionalization process and DexMA prototype fabrication of a rigid PMMA outer layer filled with DexMA hydrogel with 3.5 mm circular punch extracted from the center. **(B)** Volume comparison before (Pre) and after (Post) 10 min of near-UV crosslinking of DexMA gels (*n* = 3; mean ± s.d.; ***p < 0.001). 95% DexMA demonstrated largest volume change **(C)** Diameter change upon UV-crosslinking of DexMA gels based on image capture analysis in MATLAB **(D)** Temporal dependence of diameter change upon near-UV crosslinking of DexMA gels based on image capture analysis in MATLAB (mean ± s.d.). after 10 min of near-UV exposure. A 15–18% change in lumen diameter, necessary for physiologically relevant changes in flow, is indicated.

Similar to the prior studies, solid cylindrical hydrogels were tested for the ability to volumetrically change with response to crosslinking as a lumen coating to a PMMA ring (Figure 6). Photoinitiation was employed as the activation mechanism. The second hydrogel prototype was fabricated by methacrylation of dextran *via* glycidyl methacrylate (GMA) (van Dijk-Wolthuis et al., 1995; Trappmann et al., 2017). A round bottom flask was charged with dextran (MW = 70 kDa; 1.0 g) and 4-dimethylaminopyridine (DMAP; 0.2 g), stoppered, and purged by anhydrous nitrogen. Anhydrous DMSO (Sigma-Aldrich) was added *via* cannulation to a final concentration of 5% w/v dextran. After dissolution, glycidyl methacrylate (GMA) was injected via syringe and the reaction was carried out in the dark for 72 h at 45°C with stirring. The solution was dialyzed (Slide-A-Lyzer G2 Dialysis Cassettes, gamma-irradiated, 10 K molecular weight cutoff, Fisher Scientific) for 14 days against deionized water with dialysis media change twice a day. The DexMA solution was lyophilized until dry and stored at −20°C until use. To prepare different degrees of methacrylation (% of polymer repeat units modified), the ratio of added GMA to dextran was varied (93.0, 465.1, or 883.7 µl GMA). Three different degrees of methacrylated DexMA were achieved: 10% DexMA, 50% DexMA, and 95% DexMA.

### Geometric Change Testing of Cylindrical DexMA Hydrogels

Hydrogels of 10% DexMA, 50% DexMA and 95% DexMA were prepared from 10% w/v polymer solutions containing 0.25% w/v LAP (Sigma-Aldrich). Hydrogels (*n* = 3 per group) were photocrosslinked using a near-UV light (3D Printer UV Resin Curing Light, 405 nm, 6 W output) for 3.5 min and swollen to equilibrium in PBS, removing residual LAP. They were then moved into fresh 0.25% w/v LAP solution, subjected to photocrosslinking using the same light source, and the overall changes in volume and diameter were quantified over 10 min of light exposure. MATLAB image analysis was used to quantify the change in diameter, and to determine if the rate of diameter change was influenced by the degree of methacrylation. The volumetric change was quantified by digital caliper measurements of height and diameter pre- and post-crosslinking.

### Fabrication of DexMA Prototype

To affix the prepared DexMA to annular PMMA tubing, serving as the outer layer of the device, unmodified PMMA underwent surface plasma oxidation (1 min, PDC-32G Plasma Cleaner), followed by immediate submersion in excess methacrylic anhydride for 10 min to introduce free methacrylate groups to the surface of the PMMA tubing. After washing, the methacrylated PMMA was filled with DexMA and LAP solution (10% w/v DexMA; 0.25% w/v LAP) and photocrosslinked for 3.5 min. The center was then extracted using a 3.5 mm biopsy punch, and the device was swollen to equilibrium in PBS.

### Geometric Change Testing of DexMA Prototype

Geometric change testing of the DexMA prototype was carried out with the optimally methacrylated 95% DexMA hydrogel, which was determined through the geometric change testing of cylindrical DexMA hydrogels. After swelling the prototype to equilibrium for 24 h, another photocrosslinking event was carried out with the same near-UV light source for 10 min immediately after re-submersion in a 0.25% w/v LAP solution to assess the geometric change that occurred during near-UV exposure. The lumen expansion was quantitatively assessed by processing images taken before and after exposure using MATLAB. The outer diameter change was quantitatively assessed in the same manner to ensure that no separation from the PMMA outer layer occurred. A plot of inner and outer diameter change over time was generated and a linear regression was fit to each set of data.

### Material-Induced Hemolysis Testing

**FIGURE 7:**
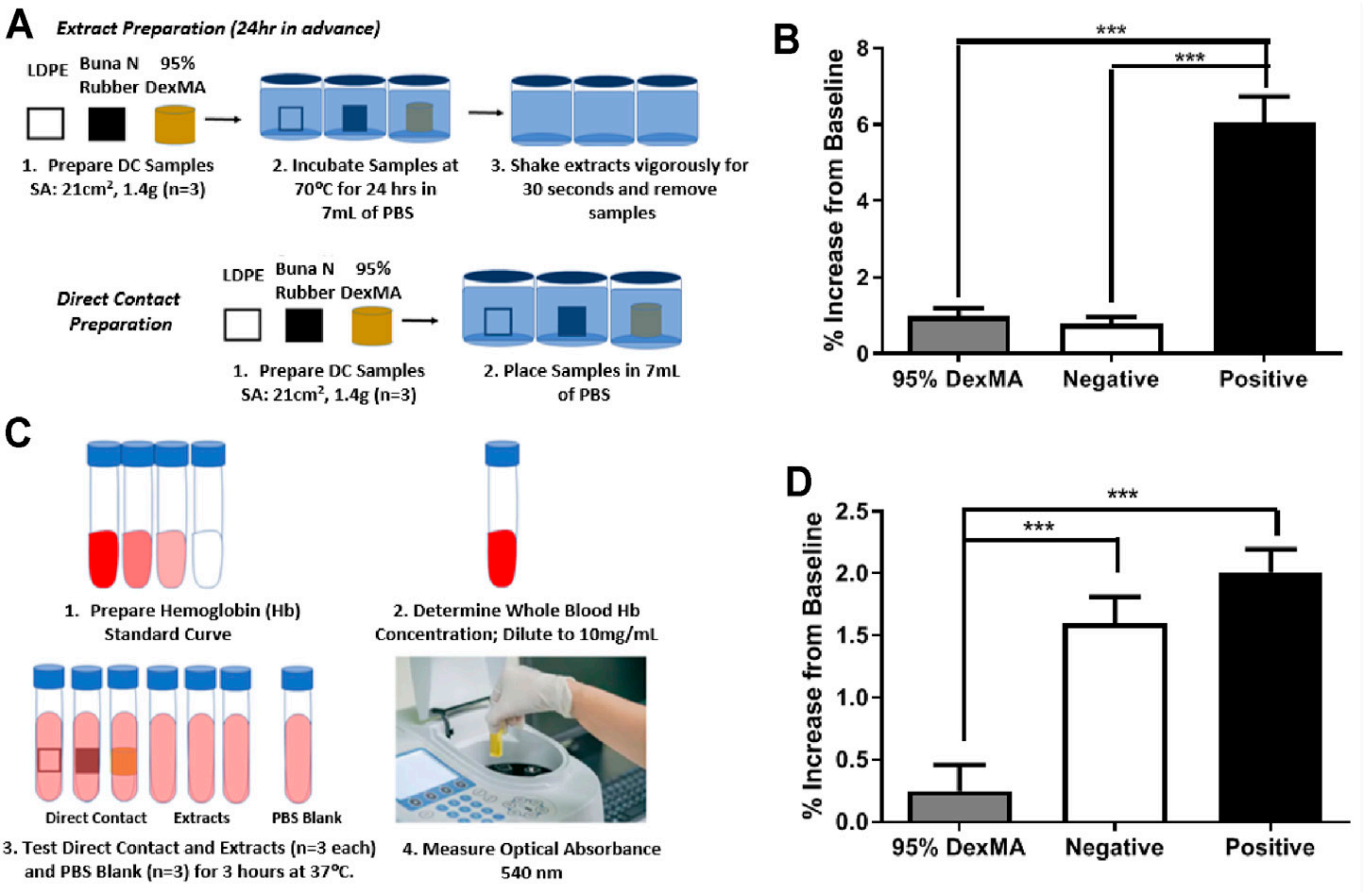
**(A)** Schematic of sample preparation for hemolytic tests. **(B)** Percent hemolysis of sample extracts, background subtracted relative to PBS (*n* = 3; mean ± s.d.; ***p < 0.001). **(C)** Schematic of hemolysis testing procedure. **(D)** Percent hemolysis of direct contact samples, background subtracted relative to PBS (*n* = 3; mean ± s.d.; ***p < 0.001).

The material-induced hemolysis of 95% DexMA was tested according to ASTM F756-17: Standard Practice for Assessment of Hemolytic Properties of Materials. The objective is to evaluate the acute hemolytic properties of the DexMA prototype when it is in contact with rabbit blood. Material extracts were prepared as specified in ASTM F619: Practice for Extraction of Medical Plastics. Relative to a positive (Buna-N-Rubber) and negative (Low-Density Polyethylene) control plastic material, the hemolytic activity of 95% DexMA (10% w/v) hydrogels was assessed and reported as specified. Figure 7 illustrates this process.

### Diameter Dimensional Analysis

To facilitate ease of fabrication, the dimensions of the gelatin prototype were larger than the shunt diameters used clinically (i.e., prototype outer diameter of 10 mm versus clinical shunt diameter of 3.5 mm). To compare the diameter change of the hydrogel prototype to the expected diameter change of an implantable shunt, a scaling relationship based on the volume was developed. Since the amount of crosslinking depends directly on the number of functional groups, the volume change between the test prototype and a theoretical implantable shunt cross-section would be the same, if the hydrogel composition does not change. Therefore, in Eq. 2, the inner diameter of the theoretical implantable shunt after crosslinking d_e,t_, was related to the starting inner diameter of the theoretical shunt, d_s,t_, and the starting and ending inner diameters of the test prototype, d_s,p_ and d_e,p_:

$$d_{e,t}=\;\sqrt{{d_{s,t}}^{2}+{d_{e,p}}^{2}-{d_{s,p}}^{2}}$$ (2)

This relationship was used to quantify the percent change in the theoretical implantable shunt in Eq. 3 based on the measured changes in diameters of the prototype:

$$\text{Δ}d_{t}=\;\frac{\sqrt{{d_{s,t}}^{2}+{d_{e,p}}^{2}-{d_{s,p}}^{2}}-d_{s,t}}{d_{s,t}}\;\times \;100$$ (3)

### Image Processing

Images of the gelatin samples were acquired using a 12-megapixel camera with a 5X digital zoom and were recorded at a fixed distance. A 2D high pass Gaussian filter was used to filter the images before processing using MATLAB (R2016b, Mathworks, Natick, MA, United States). Changes in inner lumen diameter after exposure to the crosslinking solution were measured.

### Statistical Analysis

Results are reported as the percent change (mean ± standard deviation); significance was determined by one-way analysis of variance (ANOVA) with Tukey-Kramer post-hoc testing for between group comparisons (*p* < 0.05). For paired samples before and after crosslinking, significance was assessed by paired two-tailed T-test assuming equal variance (*p* < 0.05).

## Results

### Growth Model Informs Shunt Design

To inform target design requirements, we theoretically determined a range of acceptable inner lumen diameter changes for Norwood recipients as a function of growth during shunt usage. For an ideal shunt diameter, the target clinical measure of success is to maintain a constant ratio of pulmonary to systemic blood flow, such that Qp/Qs ≤ 1.5 (Barnea et al., 1994; Austin, 1998). The cardiac outputs of healthy infants between birth and 2 years of age were used (Emmanouilides et al., 1995; Throckmorton et al., 2005). The necessary shunt flow rates, Qp, were then calculated to maintain the chosen Qp/Qs ratio of 0.9 for Norwood patients, and the results of which were modeled as a power-law function (Figure 3A) with respect to age (time in days) in Eq. 4:

$$Q_{P}\left(t\right)=0.351t^{0.118}$$ (4)

Using the power law regression and Hagen-Poiseuille relationship, we identified the requirement that the inner lumen diameter should expand by 15–18% (midpoint of 16.5%). This was used for the computational modeling and hydrogel experiments.

### Simulation Findings

Building upon the growth model, we analyzed an anatomical and computational model that was created of the shunt and Norwood physiology. In the development of this, a grid-independence study was conducted to select a mesh density that maintained the accuracy of the solution while balancing the overall simulation time. Steps were taken to ensure a high-quality mesh (y+satisfied), including refinements to satisfy the criteria of the dimensionless wall distance, the skewness, and the aspect ratio. The final model geometry consisted of approximately 6 million tetrahedral elements.

In these CFD studies, increasing inner lumen diameter was shown to increase the pulmonary-to-systemic flow (Qp/Qs) ratio, as expected, because the larger lumen diameter decreased the resistance of the shunt and allowed for more pulmonary flow. The flow ratios were found to vary between 0.6–2.6, increasing with shunt diameter (Figure 3C). The Qp/Qs ratio of the baseline model was verified to be 0.8 for the 3.5 mm shunt, reflecting distributions observed in Norwood recipients (Barnea et al., 1994).

Based on a midpoint (16.5%) of the 15–18% change range identified, a 4.08 mm model geometry was created to represent a 16.5% increase from the 3.5 mm size often used clinically. The conditions of the 4.08 mm model were applied to the 3.5 mm model to simulate the impact of growth while using a fixed shunt, the results of which showed that the Qp/Qs ratio decreased to 0.57; systemic flow dominated due to the increased cardiac output and the resistance of the narrow shunt. Higher velocities and larger regions of recirculation were found within the shunt, and more disturbed blood flow patterns were found in the pulmonary and innominate arteries.

**FIGURE 8:**
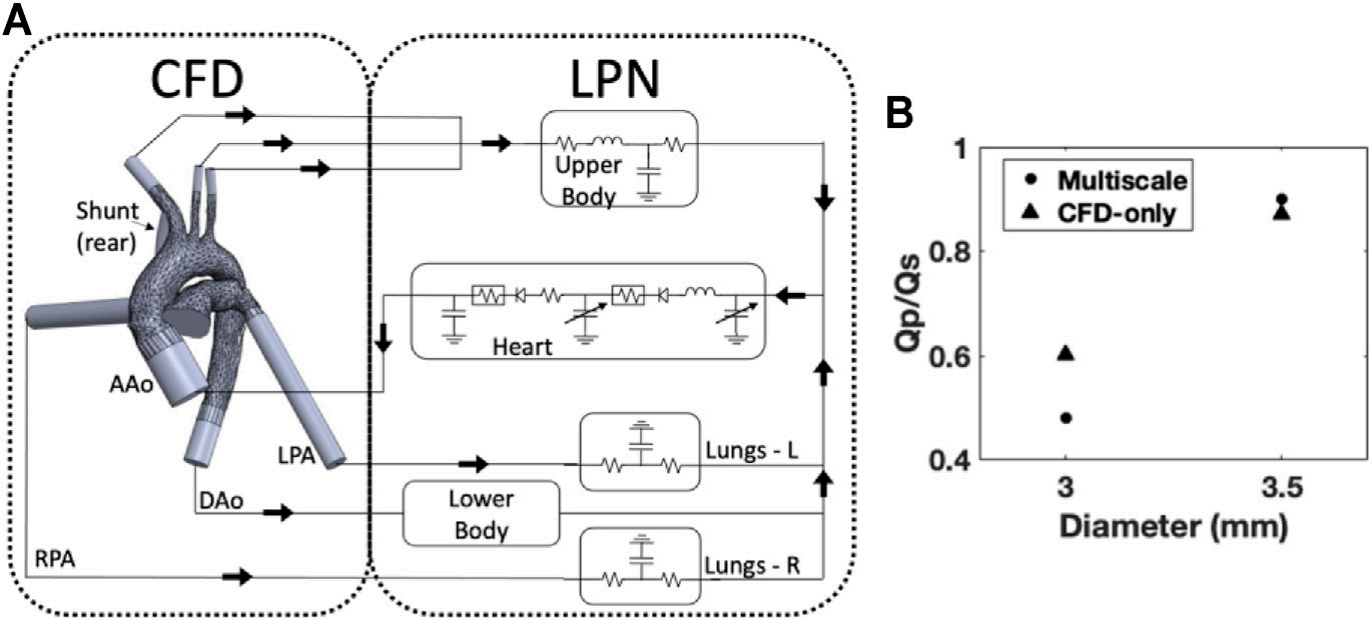
**(A)** Schematic of the multiscale model, the LPN is simplified for illustrative purposes; **(B)** The effect of diameter on the Qp/Qs ratio was reproduced for two datapoints using the multiscale model and showed similar results compared to the CFD-only approach.

The multiscale model (Figure 8A) was implemented for two diameters, 3.0 and 3.5 mm. Similar to the CFD-only approach, the parameters of the LPN were tuned to the 3.5 mm diameter and then applied to the other diameter. The resulting Qp/Qs ratios (Figure 8B) were similar to the CFD-only model and magnitudes reported in literature.

The viscoelastic blood behavior was observed through differences in the dynamic viscosity at various locations in the geometry. Shear strain rates below 100 s^−1^ were observed in regions of the fluid domain of the model. However, the entirety of the shunt was above that threshold at each hematocrit. Consequently, the dynamic viscosity was higher at the aortic inlet than in the shunt (Figure 3D). This difference was more pronounced at higher hematocrits.

### Effect of Crosslinker on Inner Diameter Expansion

The first experiment assessed the effect of the crosslinker concentration on the inner lumen diameter expansion of the hydrogel. Samples that were damaged during preparation were excluded from the analysis. All intact samples exposed to the crosslinking agent, glutaraldehyde, were observed to asymptotically increase in inner lumen diameter over the time of crosslinking, in contrast to the control group which decreased by 24 ± 5.0% due to swelling (Figures 4B–G). The rate at which the diameter changed varied between the groups. At the final timepoint (18 h), there were no significant differences observed between the crosslinked groups. The largest percent change was 16.5 ± 5.0%, in the 0.25% glutaraldehyde condition; however, the majority of the samples had a diameter change below the design target of 15–18%. Crosslinking processes had minimal effect on the outer diameter of the construct, averaging less than 0.25%. The maximum recorded change in outer diameter across all conditions was less than 1.0%, consistent with error of the image analysis methods.

### Effect of Gelatin Concentration on Inner Diameter

The effect of the gelatin concentration on the change in inner lumen diameter was assessed at two glutaraldehyde concentrations. Across both of these crosslinker concentrations, increasing gelatin concentration reduced the magnitude of the change in diameter. In the 0.05% glutaraldehyde group, the 12% gelatin sample swelled, resulting in a nearly 10% reduction in lumen diameter (Figure 5A). The largest inner lumen diameter changes occurred at 4% w/v gelatin in 0.5% glutaraldehyde, with a diameter expansion of greater than 20% (Figure 5B). The 4 and 6% gelatin samples contracted and reached the target diameter change of 15–18% in the 0.5% glutaraldehyde group. At the lower glutaraldehyde concentration, only the 4% gelatin reached the target. Final changes in diameter were highly dependent on gelatin concentration (Figures 5C,D). In the 0.05% glutaraldehyde group, all gelatin concentrations were found to differ significantly from one another. For the 0.5% glutaraldehyde case, significant differences were observed except between the 6 and 8% gelatin (*p* = 0.42), and 8 and 12% gelatin (*p* = 0.21).

### Effect of Methacrylated Dextran Concentration

Following proof-of-concept experiments with gelatin hydrogels crosslinked with glutaraldehyde, we next prepared hydrogels crosslinked with more biocompatible methods. Methacrylated dextran hydrogels were prepared and crosslinking was initiated by LAP in the presence of near-UV light. In these experiments, some degree of chemical crosslinking was required prior to initiation of the experiments, since dextran hydrogels do not crosslink physically as gelatin hydrogels do. Further increasing crosslinking via subsequent exposure to near-UV light for 10 min further decreased hydrogel volume.

A decrease in hydrogel volume was observed throughout additional near-UV exposure conducted immediately after submersion in the photoinitiator solution, dependent on the degree of methacrylation (Figure 6B). The volumetric decrease was 14.8% for the 10% DexMA, 22.1% for the 50% DexMA, and 30.5% for the 95% DexMA; all of which were significant changes from pre-exposure measurements. Interestingly, increasing methacrylation did not affect the rate of volumetric change. The slopes remained similar among data sets (0.07, 0.08, and 0.1 mm/min, respectively), but the intercept values were significantly different (i.e., initial hydrogel diameter) between each condition, reflective of different equilibrium swelling ratios prior to light exposure (Figure 6C).

### Effect of UV Exposure Time

Based on the results of this experiment and theoretical considerations, 95% DexMA was selected as the optimal formulation for continued investigation. It exhibited the largest decrease in volume upon light exposure, and should therefore allow for the greatest modulation of the shunt geometry. The 95% DexMA was therefore used for final prototype construction and evaluation of changes in lumen diameter. When evaluating the effects of light exposure time, the required 15–18% increase in lumen diameter was achieved in approximately 5 min of exposure using the 95% DexMA (Figure 6D), with a less than 1% change in the outer diameter of the prototype construct.

### First Test: Static Hemolytic Evaluation

Finally, we conducted an initial evaluation of the hemocompatibility of the dextran-based hydrogels according to ASTM F756-17, which measures hemolysis in the presence of extracts from the biomaterials or in direct contact with blood, using Buna N Rubber as a positive control. Extracts from the 95% DexMA hydrogels showed hemolytic activity comparable to negative controls (LDPE), having a hemolytic index of 1.263 (Figure 7B). In examination of direct blood contact, 95% DexMA had 30% of the hemolytic activity of LDPE (Figure 7D). This corresponds to a hemolytic index of 0.154 (nonhemolytic by direct contact test). This material is therefore suitable for blood-contacting indications, as specified by ASTM F756-17.

## Discussion

This study demonstrates the proof-of-concept that increasing crosslinking can be used to expand the diameter of hydrogel-lined shunts, with hemodynamic advantages compared to fixed-diameter shunt designs. These results provide the foundation for the development of feasible translational designs, including those with biomaterials and crosslinking agents that are safe for use in the human body [e.g., synthetic, neutral polymers crosslinked with bio-orthogonal chemistries (DeForest and Anseth, 2011)] and that can be delivered to the hydrogels by non- or minimally-invasive techniques (i.e., through the bloodstream or by release from within a delivery system contained within the hydrogel).

The design of a geometrically tunable blood shunt offers several advantages over products currently available or under investigation. Previously, researchers have attempted to address these limitations with various designs, including modifications relying on the mechanical constriction of the shunt through external plungers, balloons, ductal stenting, or clips (Douglas et al., 2010; Motohashi et al., 2018; Murtuza et al., 2012). These designs lack the ability to modify the diameter uniformly, which could increase the risk of thrombus formation. Moreover, these strategies rely on heavily invasive techniques or external equipment, which complicate their use as a durable intervention. In contrast, hydrogels crosslink relatively uniformly, and the degree of crosslinking could be controlled using minimally-invasively techniques. Additionally, previous designs have relied on data from clinical and animal studies, both of which were limited in sample sizes, and primarily neglected computational analysis despite its proven value and growing popularity in patient-specific modeling and surgical planning (Biglino et al., 2013; Arthurs et al., 2017).

In recognition of the clinical benefits of a dilatable shunt for Norwood patients, a new expandable polytetrafluoroethylene vascular conduit (PECA Labs, Inc., Pittsburgh, PA) is now available (Loneker et al., 2017; Loneker et al., 2018; Qadir et al., 2020). This device can be radially modulated via percutaneous balloon dilation and employs an expandable PTFE material of construction. Developers have demonstrated maintenance of mechanical properties post-expansion and achievement of nonhemolytic, nonpyrogenic, and noncytotoxic. In constract, we seek to advance our conduit design one step further by using external activation methods to trigger inner lumen expansion, and we are concentrating on the biomaterial chemistry and functionality in these early phase experiments.

For the repair of congenital heart defects, a number of researchers have investigated the use of tissue-engineered vascular grafts (Drews et al., 2017; Best et al., 2018). These materials were designed specifically to address the growth potential of the grafts, by replacing the artificial material used currently with a scaffold seeded to grow native tissue in its place. For the Norwood, previous studies determined that artificial grafts offered greater success in comparison to using neighboring native vessels in place of the shunt (de Leval et al., 1981; Ullom et al., 1987). These findings suggest that native vessel growth may not be reflective of the necessary hemodynamic changes in this unique anatomy; indicating that sizing of a tissue-engineered graft would also present challenges. While using tissue engineering to develop a healthy blood vessel from the patient’s own cells is attractive, these designs face substantial clinical and translational hurdles because of challenges in cell source and development of mechanical and hemodynamically functional grafts on the timescales demanded for the Norwood procedure, as well as in the manufacturing and commercialization potential of individualized grafts.

### Computational Analysis

In the computational studies, the Qp/Qs ratios in the shunt diameter study were found to agree with the relationships and magnitudes widely reported. As the diameter of the shunt increased, the pulmonary flow increased and therefore the Qp/Qs ratio increased. This behavior was expected given the fluid physics, because larger diameters have less internal resistance and therefore greater flow rates. The computational results of this study were compared with a lumped parameter model utilized in a number of published studies by Migliavacca et al. (2001). Despite the differences in approach between this study and Migliavacca et al. (2001), the recorded effect on the Qp/Qs ratio and the magnitudes of the ratio itself were in agreement, demonstrating the fundamental ability of our model to predict the hemodynamics.

Of particular interest was the effect of a 3.5–4.08 mm diameter change, which represents the specified hydrogel design requirements (15–18% increase). These results suggest that the consequences of the patient “overgrowing” the shunt include limited pulmonary flow, as well as increased regions of recirculation and other low-velocity regions, which are known to have higher thrombogenic potential (Celestin et al., 2015). These studies demonstrated the marked hemodynamic differences caused by minor geometric changes, and illustrated one of the current clinical limitations, in which even the smallest discrete diameter choices available to clinicians resulted in wide hemodynamic changes. These wide hemodynamic changes may translate into clinical consequences and poor outcomes.

The multiscale modeling effort represents the direction and intention of the next stage of computational analysis in this project. This methodology captures elements unable to be represented in CFD alone and broadens the scope of possible investigations. These techniques will be fully implemented in future work; the results of which will provide deeper insight into how the tunable shunt could function within the Norwood physiology.

The studies demonstrated that a viscoelastic blood model was successfully implemented based on the work of Good et al. (2016). There was a discrepancy in the viscosity recorded at different locations within the geometry, which is indicative of the low shear strain rates observed in the aorta. This effect was made more dramatic as the hematocrit increased, as the higher hematocrits resulted in larger viscoelastic effects. These results demonstrated the shear-thinning nature of the fluid model. In a comparison of different blood models within the aorta, Caballero and Laín (2015) found that the Newtonian assumption was sufficient for conditions with higher velocities, which supported the use of the Newtonian assumption in regions like the aortic arch. However, the regions of low shear rates observed in this model indicated that the assumption may not be reasonable. Despite the region of the shunt satisfying the Newtonian criteria, the upstream effects of low shear strain rates may alter the fluid behavior in the shunt.

Computational limitations should be noted. First, the diameter study was conducted with no other changes to the parameters of the model. Future studies should incorporate changes that may occur due to patient growth including changes in vessel shape or fluid conditions. In addition, we approximated outlet boundary conditions by applying effective pressures to achieve physiologically relevant fluid distributions, neglecting downstream peripheral resistance. This simplification was adequate for our initial comparative purposes done in steady state; however, it does limit the range of the analysis. The multiscale methodology addresses many of these limitations and will be utilized in future investigations that require more advanced modeling techniques. The viscoelastic blood model was implemented based on the work of Good et al. at only three distinct hematocrits, which constraints the range and ability of our analysis. There is no singular model of blood behavior used in the field; this is one of several models that could have been utilized. Other popular models, which have been used to describe arterial flow, include the Carreau model, the Casson model, and the Power Law model (Cho and Kensey, 1991; Johnston et al., 2004; Boyd et al., 2007). These limitations, notwithstanding, the computational models demonstrate proof-of-concept that a blood shunt with a controllable inner lumen diameter would be beneficial for patients undergoing the Norwood procedure.

While these computational findings demonstrate the critical importance of physiologically relevant boundary conditions in the modeling of these blood flow dynamics, the results supported hydrogel prototyping using the 15–18% target requirement and indicate the need for a multiphysics computational platform for this cardiovascular physiology.

### Hydrogel Prototype Development

The results of the biomaterials experiments demonstrated that changing polymer concentration and degree of crosslinking allowed control over lumen expansion to a degree that met the specified design requirements. The inner diameter expansion was studied under the effect of varying glutaraldehyde and gelatin concentrations. In the experiment investigating the effects of glutaraldehyde concentrations, higher concentrations were not statistically associated with more expansion and therefore greater amounts of crosslinking. During the period of initial exposure, higher glutaraldehyde concentrations were observed to crosslink at an increased rate, due to the initial diffusion of the crosslinker. Researchers have reported that the amount of crosslinking amplifies with increasing concentrations of glutaraldehyde, up to 1% (Bigi et al., 2001). However, it was also reported that 0.05% glutaraldehyde is sufficient to crosslink approximately 60% of the available groups.

The experimental results investigating the effects of gelatin concentrations indicated that the inner diameter expansion design requirements were achievable with lower gelatin concentrations. Hindered hydrogel contraction at increasing gelatin concentrations may be the result of polymer hydration and polymer-polymer resulsion that counter crosslink-driven deswelling. The design requirement was satisfied with a 0.5% glutaraldehyde concentration with both 4 and 6% w/v gelatin solutions, and with 0.05% glutaraldehyde with 4% w/v gelatin solutions (Figures 5, 6). While the application of glutaraldehyde crosslinking *in vivo* is limited by toxicity concerns and cross-reactivity with abundant amines in biological fluids and tissues, these studies provided evidence for the ability of tunable crosslinking approaches to achieve lumen expansion while preserving the outer diameter of the prototype.

The second prototype incorporated photocrosslinkable polymers and a potentially translational method of crosslinking via light exposure. Here, we selected methacrylated dextran (DexMA) as a base material, owing to the well-established cytocompatibility of these polymers, including the soluable form (ref) and throughout crosslinkg in the pressure of cells (ref). The Phase II hydrogel prototype achieved a physiologically relevant lumen diameter increase within minutes of near-UV exposure. The device is therefore capable of achieving the dimensional changes needed to support patient growth while remaining affixed to the outer layer. Furthermore, DexMA is nonhemolytic under ASTM F756-17 and is suitable for the blood-contacting indication that this design addresses. LAP is a non-toxic and water-soluble photoinitiator with a broad activation spectrum spanning the UV and near-UV range (Fairbanks et al., 2009), which could be used to achieve crosslinking in a methacrylated hydrogel *in vivo*. Though, the methods by which the photoinitiator is introduced into the hydrogel lumen may require ongoing refinement. Studies here have demonstrated that additional photocrosslinking is possible immediately after introduction of LAP in a static environment. Outcomes are likely the result of rapid LAP diffusion, owing to the high degree of water solubility, in combination with radical diffusion mediated by both physical and chemical processes (Colvin et al., 1997; Higgins et al., 2020). Given the permissive route for photoinitiator introduction, percutaneous catheter placement inside of the shunt could feasibly be used to introduce soluble LAP concurrent with exposure to near-UV light *via* a fiber-optic light guide. These methods, however, necessitate ongoing development of suitable light guide assemblied to perform the procedure and investigation of the methods under flow that better reflects *in vivo* conditions.

Static blood-contact hemocompatibility studies were performed on the DexMA shunt prototype. The rationale for this static evaluation stems from these studies being a reasonable first step evaluation in the efficacy of using DexMA for this application. We recognize the importance of experiments under more physiologic and shear flow conditions. It is important to assess how well these hydrogels will retain their structure, function and stability during blood flow conditions. Shunt thrombosis is of high occurrence in the currently used shunts, thus is a demonstrated mortality and morbidity risk for these patients. This new hydrogel-lined conduit must incorporate material properties that mitigate platelet adhesion and triggers leading to coagulation. These will be a priority in forthcoming investigations.

Limitations in manufacturing techniques must also be considered as we plan to construct these shunts with longer lengths. General clinical translation of this approach requires the development of responsive hydrogel systems that are compatible with the blood-contacting environment and which leverage bio-orthogonal crosslinking chemistries. As a blood-contacting biomaterial, the shunt should also be subjected to rigorous hemocompatibility testing, including for toxicity and thrombogenicity in the next phase of development.

## Conclusion

A novel tunable shunt design offers a new treatment paradigm to rebalance blood flow in neonatal patients who receive the complex Norwood procedure and who seek cardiophysiologic stabilization that accompanies growth and development post-operatively. The experimental and computational results of this investigation have demonstrated proof-of-concept and feasibility in the design of a geometrically tunable hydrogel-based shunt. The growth model and computational cardiovascular studies informed the design requirement for the inner lumen diameter change (15–18%) and revealed the importance of physiologically relevant boundary conditions in the modeling of these blood fluid dynamics. The hydrogel experimental results tested and demonstrated achievement of the target design requirement for inner lumen diameter change. The satisfaction of this requirement is a positive indicator of the design feasibility going forward. DexMA is nonhemolytic under ASTM F756-17 and is suitable for the blood-contacting indication that this device treats. Taken together these studies are parallel and complementary endeavors that demonstrated the feasibility of a new device design and advanced the development effort of an innovative geometrically tunable blood shunt for Norwood recipients.

## Data Availability

The raw data supporting the conclusion of this article will be made available by the authors, without undue reservation.
